# How EF-Tu can contribute to efficient proofreading of aa-tRNA by the ribosome

**DOI:** 10.1038/ncomms13314

**Published:** 2016-10-31

**Authors:** Jeffrey K. Noel, Paul C. Whitford

**Affiliations:** 1 Center for Theoretical Biological Physics, Rice University, Houston, Texas 77030, USA; 2 Max Delbrück Center for Molecular Medicine, Kristallographie, Robert-Rössle-Strasse 10, Berlin 13125, Germany; 3 Department of Physical Chemistry, Fritz Haber Institute of the Max Planck Society, Berlin 14195, Germany; 4 Department of Physics, Northeastern University, Dana Research Center 111, 360 Huntington Avenue, Boston, Massachusetts 02115, USA

## Abstract

It has long been recognized that the thermodynamics of mRNA–tRNA base pairing is insufficient to explain the high fidelity and efficiency of aminoacyl-tRNA (aa-tRNA) selection by the ribosome. To rationalize this apparent inconsistency, Hopfield proposed that the ribosome may improve accuracy by utilizing a multi-step kinetic proofreading mechanism. While biochemical, structural and single-molecule studies have provided a detailed characterization of aa-tRNA selection, there is a limited understanding of how the physical–chemical properties of the ribosome enable proofreading. To this end, we probe the role of EF-Tu during aa-tRNA accommodation (the proofreading step) through the use of energy landscape principles, molecular dynamics simulations and kinetic models. We find that the steric composition of EF-Tu can reduce the free-energy barrier associated with the first step of accommodation: elbow accommodation. We interpret this effect within an extended kinetic model of accommodation and show how EF-Tu can contribute to efficient and accurate proofreading.

## Abstract (web summary)

The translation of mRNA by the ribosome is governed by a series of large-scale conformational transitions. Here the authors use MD simulations to demonstrate how the rate of dissociation of elongation factor Tu affects the dynamics of tRNA accommodation and proofreading.

The ribosome is a large RNA-protein assembly (2–3 MDa) and it is the sole producer of proteins in the cell. The elongation process, in which messenger RNA (mRNA) is read and proteins are synthesized, involves multiple large-scale conformational transitions that result in individual amino acids being added to the growing protein chain^1^,^2^,^3^,^4^,^5^,^6^,^7^. Each round of elongation begins with the delivery of an aminoacyl-transfer RNA (aa-tRNA) molecule to the ribosome by elongation factor thermally unstable (EF-Tu in bacteria, or eEF1A in eukaryotes). During initial association of ternary complex (aa-tRNȦEF-Tu̇GTP) with the ribosome, codon–anticodon interactions are formed between aa-tRNA and mRNA. Based on differences in hydrogen bonding energies, a ternary complex that contains a cognate (correct) aa-tRNA molecule is only marginally more stable (several *k*_B_*T*) than one carrying a near-cognate aa-tRNA. Such small differences in stability can explain a preference for correct, over incorrect, tRNAs of approximately 100:1. However, in bacteria, the accuracy of translation is roughly 3,000:1, or greater (for review, see refs 1, 7). To account for this discrepancy, Hopfield showed that the fidelity of aa-tRNA selection could be amplified if it were composed of at least two steps that are separated by an irreversible chemical event^8^.

Decades of biochemical, structural and single-molecule measurements have provided a broad understanding of the tRNA selection process. The existence of proofreading was originally verified by biochemical data that implicated two distinct steps during tRNA selection (that is, initial selection and proofreading)^9^,^10^. During initial selection, aa-tRNA (in complex with EF-Tu·GTP) forms codon–anticodon interactions with mRNA that trigger GTPase activation and finally hydrolysis^11^,^12^,^13^. GTP hydrolysis is followed by Pi release, which signals a conformational change in EF-Tu (ref. 14) and aa-tRNA accommodation (the proofreading step). The observed level of proofreading is typically between 15 and 60 (refs 9, 10, 12, 13, 15, 16, 17, 18; though larger values have been reported^19^), where variations may arise from differences in experimental protocols and mismatch composition. From a structural perspective, cryoelectron microscopy (cryo-EM) and X-ray crystallography have elucidated the conformational properties of these biochemically identified states and have provided detailed insights into steps preceding and following accommodation^20^,^21^,^22^,^23^,^24^,^25^,^26^. To complement these static snapshots, single-molecule FRET (smFRET) studies have shed light on the dynamics of individual aa-tRNA molecules during initial selection^27^ and accommodation^28^. This body of work provides a strong structural and biochemical foundation, upon which our physical–chemical understanding may be refined.

Experimental studies have forcefully demonstrated that proofreading is associated with aa-tRNA accommodation, though the role of EF-Tu during this process is less clear. Accommodation involves a large conformational rearrangement (≈100 Å) that follows Pi release and precedes peptide bond formation, where kinetic partitioning at this step can account for observed levels of proofreading^12^,^13^. Consistent with a large rearrangement being necessary for accurate selection, subtle mutations to the aa-tRNA can perturb^29^ the A/T conformation (Fig. 1b) and impact the degree of proofreading^30^,^31^. With regards to the molecular factors that govern selectivity, biochemical studies have identified many important inter- and intra-molecular interactions^17^,^32^ and have shown that the same molecular features may be exploited during initial selection and proofreading^15^. However, the latter observation appears to be sensitive to the tRNA species, which suggests the possibility of multiple proofreading mechanisms^19^. Despite some remaining controversies (for review, see ref. 7), it is widely accepted that a delicate balance of kinetic steps is critical for accurate proofreading. In the prevailing kinetic model of proofreading, aa-tRNA accommodation and EF-Tu dissociation from the ribosome are treated as two independent and parallel steps^1^,^12^,^13^,^15^,^17^,^18^,^19^,^30^,^32^,^33^. Since they occur on comparable timescales^14^,^33^,^34^, the current study explores the physical relationship between these potentially inter-related events.

Computational^35^,^36^ and experimental^28^,^37^,^38^ studies have implicated the following sequence of conformational rearrangements during aa-tRNA accommodation: (1) aa-tRNA moves from an A/T to an elbow-accommodated (EA) conformation, (2) the aa-tRNA arm accommodates into the A-site and (3) the 3′-CCA end enters the peptidyl transferase centre (PTC). The first simulations of factor-free accommodation employed targeting techniques and showed that sequential movement of the elbow, arm and 3′-CCA end is sterically accessible^35^. Unrestrained simulations that used an electrostatics-free model corroborated this overall ordering of events and predicted that the EA conformation represents an intermediate (that is, a local free-energy minimum)^36^. smFRET measurements have shown that the incoming aa-tRNA may reversibly sample EA-like and A/T-like ensembles before peptide bond formation occurs^28^, consistent with elbow accommodation preceding 3′-CCA accommodation. Other experiments have found that mischarging aa-tRNA (attaching the wrong amino acid) does not alter elbow dynamics^37^, which suggests elbow and 3′-CCA accommodation are separable events. Finally, a cryo-EM reconstruction of the ribosome in complex with the antibiotic HygA found the elbow to be in an accommodated position, while the 3′-CCA end was displaced from the A-site^38^. The same study also applied smFRET and reported the signature of an intermediate in which the elbow is nearly fully accommodated, which would appear to be consistent with the predicted EA intermediate^36^. Taken together, these theoretical and experimental studies implicate a multi-step accommodation process that begins with reversible elbow movement into the A-site and finishes with 3′-CCA end accommodation into the PTC.

Through the use of energy landscape principles^39^,^40^ and molecular dynamics simulations, we provide evidence for how the rate of EF-Tu dissociation can affect accommodation dynamics and proofreading. By employing a model in which all non-hydrogen atoms are represented, these simulations highlight the large influence steric interactions can have on the free-energy landscape of accommodation. Specifically, we find that the steric composition of EF-Tu allows it to increase the rate of elbow accommodation by several orders of magnitude. While we find a conformational change in EF-Tu can amplify this effect, it is not required. These observations show how EF-Tu may drive aa-tRNA into a partially accommodated conformation, which provides a molecular-level physical–chemical explanation for how EF-Tu may help facilitate efficient proofreading by the ribosome.

## Results

### An energetic model for aa-tRNA accomodation

There are many force fields available for the simulation of biomolecules, each suited to address different aspects of the dynamics. For example, explicit-solvent models provide a detailed description of RNA and protein energetics, which has enabled the simulation of folding of tetraloops^41^,^42^ and the calculation of free-energies for isolated regions of the ribosome^43^,^44^, as well as the evaluation of enthalpies^45^ and diffusion coefficients^46^ for fully-assembled ribosomes. While these models have proven to be effective when describing small-scale/narrowly defined conformational transitions, the vast phase space associated with accommodation makes it computationally intractable to calculate the free energy. In contrast, through the use of simpler models, one may evaluate and compare the free energy of accommodation for a variety of molecular constructs. In the current study, we adopt this approach and perform simulations using an all-atom structure-based model (SBM)^47^,^48^ constructed from a structural model of the ribosome in complex with aa-tRNA^Phe^ (ref. 25). This allows us to identify how the shape of the ribosome and EF-Tu contribute to the free-energy landscape of elbow accommodation (the first substep of accommodation).

**Figure 1:**
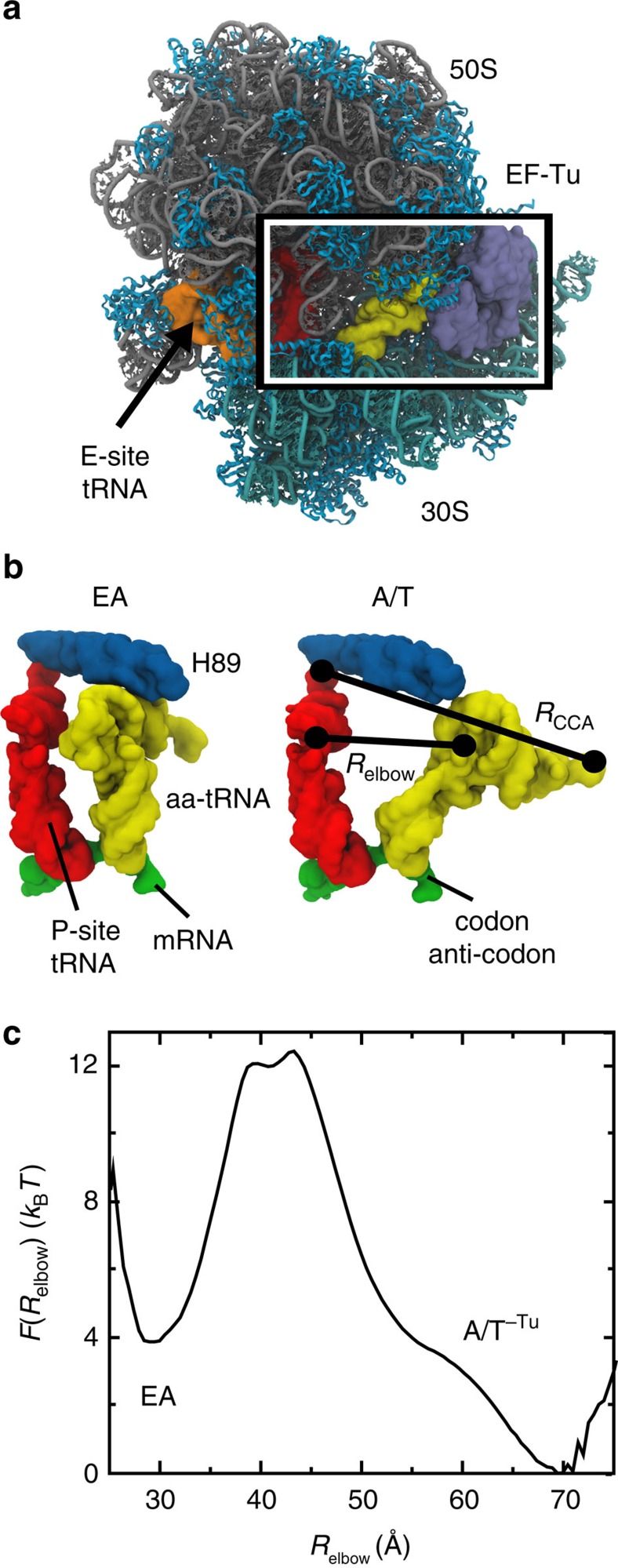
aa-tRNA elbow accommodation has a two-state-like free-energy landscape. (**a**) Full 70S ribosome^29^ with 23S rRNA in grey, 16S rRNA in cyan and proteins in blue. The A/T-configured aa-tRNA is shown in yellow. P and E-site tRNAs are shown in red and orange. EF-Tu is shown in ice blue. The white/black box outlines the accommodation corridor^35^. (**b**) The first step of aa-tRNA accommodation involves elbow movement, where it transitions from the A/T to EA conformation and partially binds the ribosomal A-site. *R*_elbow_ is the distance between the O3′ atoms of U8 in the P-site tRNA and U60 of aa-tRNA^48^. *R*_CCA_ (separation of O3′ atoms of A76 in the aa-tRNA and P-site tRNA) monitors movement of the 3′-CCA end towards the peptidyl transferase centre. (**c**) With the employed model, the free energy as a function of *R*_elbow_ possesses two dominant minima, which correspond to EA and A/T-like (labelled A/T^−Tu^) ensembles. The barrier arises from steric interactions between aa-tRNA and H89 (Supplementary Fig. 2). All structural depictions were prepared using VMD^70^.

In the model used here, all non-hydrogen atoms are explicitly represented, and interatomic interactions specifically stabilize the A/A conformation (the endpoint of accommodation, Fig. 1b). This construction of the potential energy is referred to as a structure-based model (SBM) since the minimum is defined by a preassigned structure. In this class of models, electrostatic and solvent contributions are typically accounted for implicitly, where each stabilizing interaction describes the net effect of the myriad energetic factors that favour the experimentally obtained structure^49^. In addition to the purely structure-based interactions, we also include explicit short-range electrostatic (Debye-Hückel) interactions between the tRNA and accommodation corridor, as employed in protein-DNA^50^ and protein folding^51^ simulations. The rationale for including these non-specific interactions (that is, those not found in the A/A structure) is that the aa-tRNA molecule samples conformations far from A/A during accommodation. As discussed below, we find that the magnitude of the free-energy barrier depends on the electrostatic details. However, qualitative similarities between the free-energy profiles obtained with electrostatic and electrostatics-free models (Supplementary Fig. 1) suggest that biomolecular structure is a major determinant of the overall character of elbow accommodation.

### Kinetics and free energy of factor-free accommodation

With the employed forcefield, the free-energy profile associated with aa-tRNA elbow accommodation has two clear minima that are separated by a significant free-energy barrier (Fig. 1c). The minima correspond to EA (*R*_elbow_∼30 Å) and extended (A/T-like, *R*_elbow_∼70 Å) ensembles. We will refer to the extended ensemble as A/T^−Tu^, since EF-Tu is not included in this calculation. In the A/T^−Tu^ basin, aa-tRNA is base paired with mRNA, and it can adopt larger values of *R*_elbow_ than found in A/T crystal structures^24^,^29^. For a more detailed description of the A/T ensemble, see ref. 36. Note that, since the current calculations only sampled the A/T to EA transition, and not 3′-CCA end accommodation, the EA free-energy minimum is shifted slightly from the A/A value of *R*_elbow_^36^.

To describe the free-energy barrier associated with elbow accommodation, one may inspect the structural properties of the transition state ensemble (TSE)^52^,^53^,^54^. In the current model, steric interactions lead to displacement of H89 in the TSE (Supplementary Fig. 2c), consistent with the transient formation of H89-tRNA interactions^35^,^48^ and previous predictions of H89 movement^36^. Experimentally, mutations in the vicinity of H89 have been found to affect accommodation^55^, which also suggests H89-tRNA interactions may be formed in the TSE. To verify that the barrier arises from H89 interactions, we repeated our calculations using a model in which H89-tRNA interactions are absent. Upon making this virtual deletion of H89, the free-energy barrier is largely attenuated (Supplementary Fig. 2a,b). These observations highlight how the ribosome can impose strict boundaries on the available aa-tRNA conformations, which lead to this sterically induced free-energy barrier.

To calculate rates from free-energy profiles, we describe elbow accommodation in terms of diffusive movement across a free-energy landscape^39^,^52^,^56^. Specifically, rates are calculated via the relation^39^,^57^:


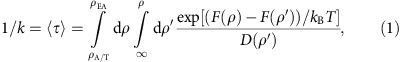


where *ρ* is the reaction coordinate *R*_elbow_ (the distance between U60 in aa-tRNA and U8 in the P-site tRNA) and *D* is the diffusion coefficient (estimated from explicit-solvent simulations^46^). While interatomic distances can provide intuitive descriptions of elbow movement, the full dynamics are multi-dimensional. To ensure that the essential characteristics of the process are not masked by the choice of coordinate^58^, we previously showed that *R*_elbow_ can reliably separate the end points and accurately capture the TSE associated with elbow accommodation^48^. Further, motion along *R*_elbow_ was observed to be diffusive in the neighbourhood of the TSE. Accordingly, one may use equation (1) to calculate rates from free-energy profiles. As an example, the barrier in Fig. 1c would correspond to a rate of ∼20 s^−1^ for EF-Tu-free elbow accommodation, which is similar to experimentally determined rates for full accommodation (∼40 s^−1^ (ref. 16)). The similarity of these numbers suggests that the theoretical free-energy barriers are within a biologically relevant kinetic regime, though the quantitative relationship between biochemically measured rates and the elbow accommodation substep has not been rigorously established.

### EF-Tu can accelerate elbow accommodation

**Figure 2:**
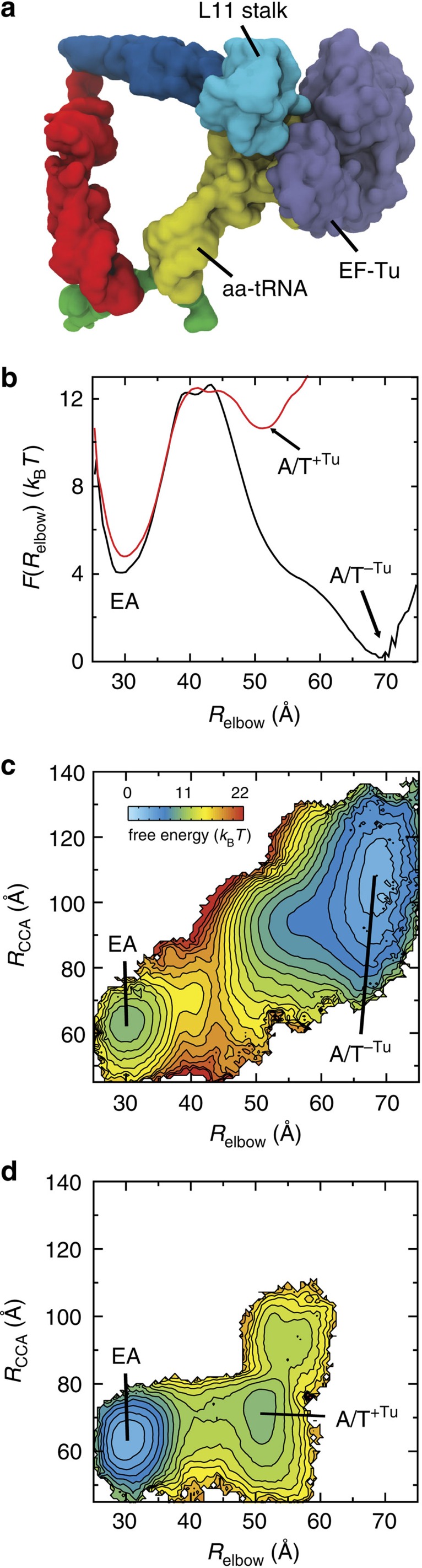
EF-Tu occludes access of aa-tRNA to A/T-like conformations. (**a**) EF-Tu, in the form of ternary complex (EF-Tu̇GTṖaa-tRNA), binds the ribosome at the opening of the accommodation corridor^20^,^21^,^22^,^23^,^24^,^26^,^29^. (**b**) When EF-Tu is restrained to its GTP conformation on the ribosome, and a minimal affinity for aa-tRNA is included, the barrier to elbow accommodation is reduced by ∼10 *k*_B_*T* (red curve), relative to the barrier when EF-Tu is absent (black curve). (**c**,**d**) Two-dimensional free-energy profiles, calculated for the models that lack EF-Tu (**c**) and include EF-Tu (**d**). There is a drastic reduction in the range of accessible conformations of aa-tRNA when EF-Tu is bound to the ribosome. This leads to destabilization, and shift in position, of the A/T-like ensemble (A/T^−Tu^ in **c** and A/T^+Tu^ in **d**).

Inspired by the experimental observation that aa-tRNA accommodation and EF-Tu dissociation occur on comparable timescales^14^,^33^,^34^, we asked whether EF-Tu can directly influence the rate of accommodation after it releases the tRNA molecule. To this end, we used our simplified model to calculate the free energy of aa-tRNA elbow accommodation with EF-Tu bound to the ribosome in its post-hydrolysis, pre-Pi-release conformation^21^,^29^. Relative to the calculations described in Fig. 1, the only difference in this set of simulations is that EF-Tu is restrained to a ribosome-bound conformation^29^: Tu^A/T^ (Fig. 2a). We find that the presence of EF-Tu shifts the A/T basin by 15 Å, from *R*_elbow_≈70 Å to ≈55 Å (Fig. 2b). In this implementation of the model, EF-Tu and aa-tRNA only interact through non-specific electrostatic and excluded-volume interactions. Despite the fact that these interactions slightly favour A/T conformations (Supplementary Fig. 3), the A/T ensemble is destabilized by more than 10 *k*_B_*T* (6 kcal mol^−1^, Fig. 2b). One may interpret this as arising from a reduction in the number of conformations that are accessible to the tRNA molecule when EF-Tu is present. That is, many A/T-like conformations are sterically occluded by the presence of EF-Tu (Fig. 2d), where more extended conformations are only accessible when EF-Tu is absent (Fig. 2c). An interesting observation is that, while EF-Tu significantly affects the stability of the A/T ensemble, the location of the free-energy barrier (*R*_elbow_≈40 Å and *R*_CCA_≈70 Å) is consistent with that observed in the EF-Tu-free simulations. This indicates that, rather than change the dominant route of accommodation, EF-Tu helps aa-tRNA overcome this pre-existing barrier.

The decrease in barrier height when EF-Tu is bound raises the possibility that elbow accommodation is accelerated by EF-Tu. In accordance with the discussion in the previous section, we used equation (1) to calculate the predicted rate of elbow accommodation in the presence of EF-Tu. The profiles in Fig. 2b would implicate the following rates for cognate aa-tRNA: 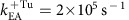, 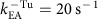, 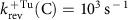 and 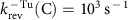, where (C) indicates that a rate is associated with cognate aa-tRNA and the subscript rev denotes reverse-elbow accommodation. It is important to recognize that, while 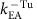 is comparable to experimentally reported rates of full accommodation^16^, the scale of the barrier can be influenced by minor changes in the electrostatic model. Accordingly, the overall magnitude of the rates should not be interpreted as a precise prediction. Instead, the principal conclusion one should draw is that the rate of elbow accommodation can be increased significantly (>1,000-fold) if EF-Tu releases aa-tRNA before dissociating from the ribosome.

### Robustness of EF-Tu-accelerated elbow accommodation

**Figure 3:**
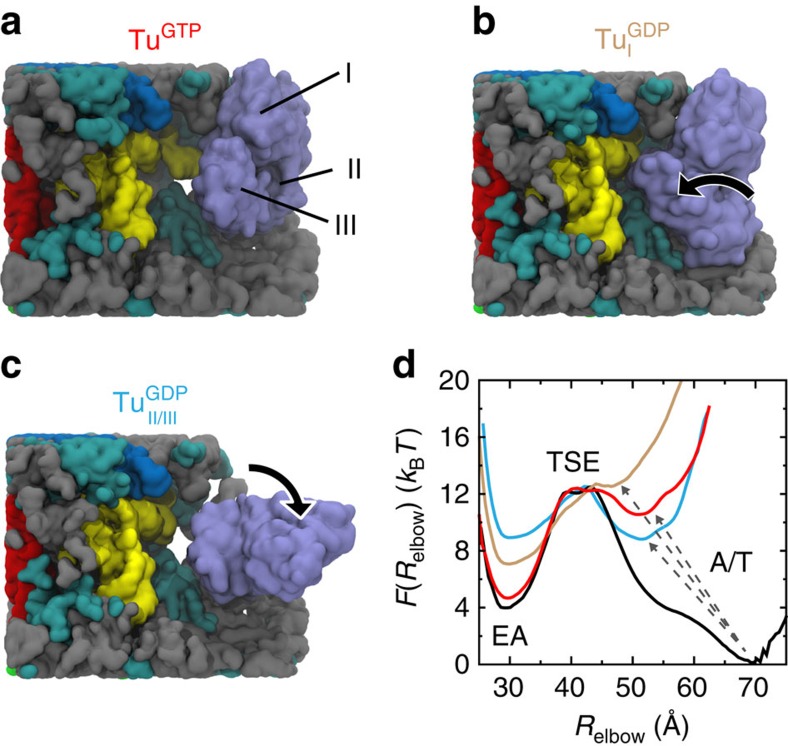
The steric influence of EF-Tu on accommodation is robust. (**a**) EF-Tu in a pre-Pi-release conformation, aligned to the simulated system. In this conformation, domain I of EF-Tu contacts the 50S subunit, while domains II/III contact protein S12 and the neighbouring RNA on the 30S subunit. Upon Pi release, EF-Tu undergoes a conformational transition while still associated with the ribosome^33^, where at least one point of contact is likely to be maintained. To probe the influence of EF-Tu·GDP on accommodation, we constructed hypothetical structural models, where EF-Tu·GDP is aligned to either (**b**) domain I 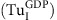, or (**c**) domains II/III 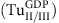 of the EF-Tu·GTP structure. In panels **a**–**c**, RNA and proteins in the accommodation corridor are coloured gray and cyan, and the remaining molecules are coloured as in Fig. 2. The L11 stalk is not visualized, though it was included in all simulations. (**d**) For all models, the A/T basin is significantly destabilized (arrows) when EF-Tu is present. In contrast to the shift in position of the A/T-like basin, the locations of the EA basin and the TSE are insensitive to the conformation of EF-Tu.

To further characterize the role of EF-Tu sterics on accommodation, we considered additional conformations that EF-Tu may adopt before it dissociates from the ribosome. While it is known that the formation of codon–anticodon interactions triggers GTP hydrolysis, which leads to Pi release and a conformational change in EF-Tu^11^,^12^,^13^, the precise role of this large-scale rearrangement is not fully understood. Accordingly, we constructed several structural models of potential post-Pi-release configurations, to determine whether EF-Tu-accelerated elbow accommodation is robust to the conformation of EF-Tu. In the pre-Pi-release conformation of ternary complex^20^,^21^,^22^,^23^,^24^, the 3′-CCA end of aa-tRNA binds to a crevice between domains I and II of EF-Tu. This interface is not formed in the GDP form of EF-Tu (ref. 59), consistent with its reduced affinity for aa-tRNA^60^. In addition, in the GDP form, there is a displacement of domain I and the II/III superdomain (Fig. 3a–c), relative to the GTP form. To study the effects of such a rearrangement on accommodation, we considered two possible orientations of EF-Tu·GDP^59^, where it was partially aligned (see the ‘Methods' section) to the ribosome-bound GTP conformation of EF-Tu (ref. 29). In the first scenario, interactions between domain I and the ribosome remain intact, while domains II/III are displaced (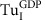, Fig. 3b). Alternately, the interface between domains II/III and the ribosome may remain formed, while domain I pivots (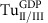, Fig. 3c).

To assess the kinetic consequences of a structural rearrangement in EF-Tu before dissociation from the ribosome, we recalculated the free-energy profiles for accommodation with EF-Tu in the 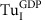, or 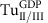, configuration (Fig. 3d). In the 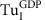 configuration, domains II/III are displaced towards the ribosomal A-site (relative to Tu^A/T^), in a manner that would resemble a power-stroke-like conformational change. This configuration of EF-Tu precludes sampling of the A/T-like ensemble and yields a free-energy profile that is ‘downhill' in the direction of accommodation (Fig. 3d, tan curve). In the 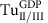 configuration, domain I is displaced away from the ribosomal A-site. While this represents a markedly different orientation of EF-Tu, the 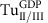 configuration also significantly destabilizes the A/T ensemble (Fig. 3d, cyan curve). This demonstrates that the effect of EF-Tu on accommodation is robust to the precise sequence of rearrangements that may occur after EF-Tu releases the tRNA molecule. Specifically, given that EF-Tu remains nominally associated with the ribosome, the elbow accommodation barrier should be significantly reduced. While the precise value of the barrier depends on the conformation, we find that EF-Tu destabilizes the A/T basin by at least 8 *k*_B_*T* for all post-hydrolysis models, relative to when EF-Tu is absent. Since the rate is exponentially related to the barrier height, these results reinforce the finding that elbow accommodation may be accelerated by EF-Tu (by a factor of *e*^8^∼10^3^). This sterics-based mode of acceleration is distinct from other assemblies that directly utilize chemical energy to overcome a rate-limiting barrier^61^,^62^. That is, these results implicate an indirect mode of coupling between hydrolysis on EF-Tu and accommodation dynamics, though a power stoke may amplify the effect.

### An extended kinetic model for proofreading

**Figure 4:**
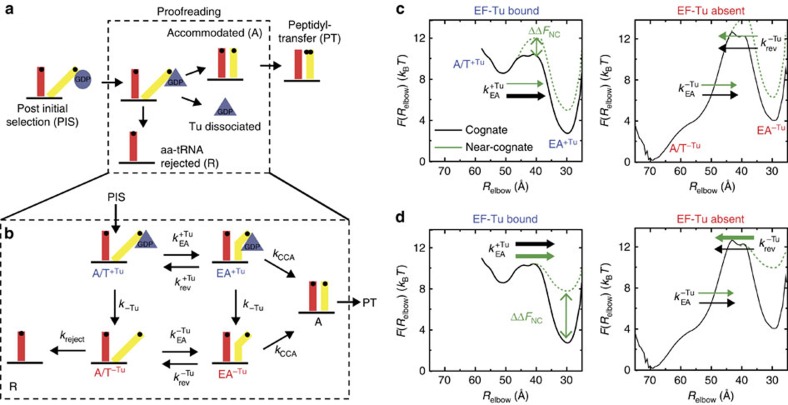
Simulated landscapes suggest a kinetic model for proofreading that includes the influence of EF-Tu on elbow accommodation. (**a**) Schematic representation of the current model of proofreading^12^,^13^,^15^,^17^,^18^,^19^,^30^,^32^,^33^. After initial selection is completed (PIS), accommodation, EF-Tu dissociation and rejection are described as parallel kinetic steps. (**b**) An extended kinetic model for accommodation suggested by simulations. After initial selection, aa-tRNA will reach one of two states: accommodated (A) or rejected (R). The top branch (EF-Tu bound) transitions to the bottom branch (EF-Tu absent) at the rate of *k*_−Tu_. (**c**) The free-energy profiles describing the transition between A/T and EA conformations are shown for EF-Tu bound (left) and EF-Tu absent (right). An induced fit mechanism of accommodation would imply an increased barrier height for near-cognate tRNA (ΔΔ*F*_NC_). In terms of the extended kinetic model, this would correspond to smaller values for 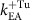 and 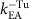 for near-cognate molecules, while leaving 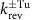 unchanged. (**d**) An alternative potential effect of near-cognate codon interactions is that the EA ensemble is destabilized. In that scenario, the forward rates would be identical for all tRNA molecules, whereas the reverse rates would change. To explore the impact of EF-Tu dissociation on fidelity, the proofreading factor and efficiency were calculated for each of these energetic descriptions of near-cognate molecules.

Biochemical analysis has provided an intricate kinetic description of the elongation process^12^,^13^,^15^,^17^,^18^,^19^,^30^,^32^,^33^,^63^. With the current calculations suggesting that EF-Tu may influence the rate of accommodation, we have extended the prevailing kinetic model for proofreading (Fig. 4a) to include substates that account for the relationship between EF-Tu and accommodation (Fig. 4b). The essential features of this extended model are: (1) significant free-energy barriers are associated with aa-tRNA elbow accommodation and 3′-CCA end accommodation^36^. (2) aa-tRNA can reversibly sample EA-like conformations^28^. (3) The rate of forward elbow accommodation is accelerated by EF-Tu̇GDP. (4) EF-Tu̇GDP dissociation from the ribosome is irreversible. One should note that allowing for re-association of EF-Tu̇GDP is kinetically equivalent to decreasing the effective rate of dissociation (*k*_−Tu_), which does not qualitatively change the presented analysis. (5) We assume that a rejected aa-tRNA follows the same path, but in the opposite direction, as an accepted aa-tRNA. As a consequence, aa-tRNA may only be rejected after EF-Tu dissociates from the ribosome. (6) 3′-CCA end accommodation is treated as irreversible. This assumption is supported by the fact that it is possible to crystallize the A/A conformation for both cognate and near-cognate tRNA^64^, indicating that the A/A conformation can be very stable. This set of conditions suggests a kinetic model for accommodation that consists of the following six states (Fig. 4b): aa-tRNA may adopt an A/T-like conformation (post initial selection) with EF-Tu bound (A/T^+Tu^) or without EF-Tu bound (A/T^−Tu^) to the ribosome; aa-tRNA may adopt an EA conformation in the presence of EF-Tu (EA^+Tu^), or in the absence of EF-Tu (EA^−Tu^); aa-tRNA may be fully accommodated (A) or rejected (R).

The extended kinetic model (Fig. 4b) was used to evaluate the selectivity of proofreading, as well as the efficiency for a range of kinetic parameters. These calculations specifically focused on the proofreading factor *P*_f_. Proofreading contributes to the total selectivity of translation according to *I* × *P*_f_, where *I* is the selectivity of initial selection. To calculate *P*_f_, we first derived expressions for the rates of rejection and accommodation under the steady-state approximation, which entails analysing the populations of states A and R, given a fixed population of the A/T^+Tu^ state. This provides the probability that aa-tRNA will be accepted (A) or rejected (R) after successfully passing initial selection (labelled PIS in Fig. 4). *P*_f_ is defined as the ratio of cognate ([A]^C^) to near/non-cognate ([A]^NC^) tRNA that arrive at the fully accommodated state:


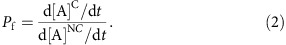


The efficiency *E* of proofreading is then defined as the number of cognate tRNA molecules that are accommodated, relative to the total number of cognate tRNA molecules that pass initial selection:


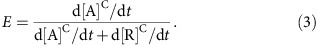


For a detailed discussion of the kinetic equations, see Supplementary Methods. Below, we consider two different ways in which the landscape may depend on cognate and near-cognate interactions. For both types of landscape effects, we calculated the corresponding proofreading factor and efficiency for a wide range of EF-Tu dissociation rates. This analysis suggests how previously measured levels of proofreading may depend, in part, on the precise timing of EF-Tu release from the ribosome.

### EF-Tu and the efficiency of induced fit based proofreading

**Figure 5:**
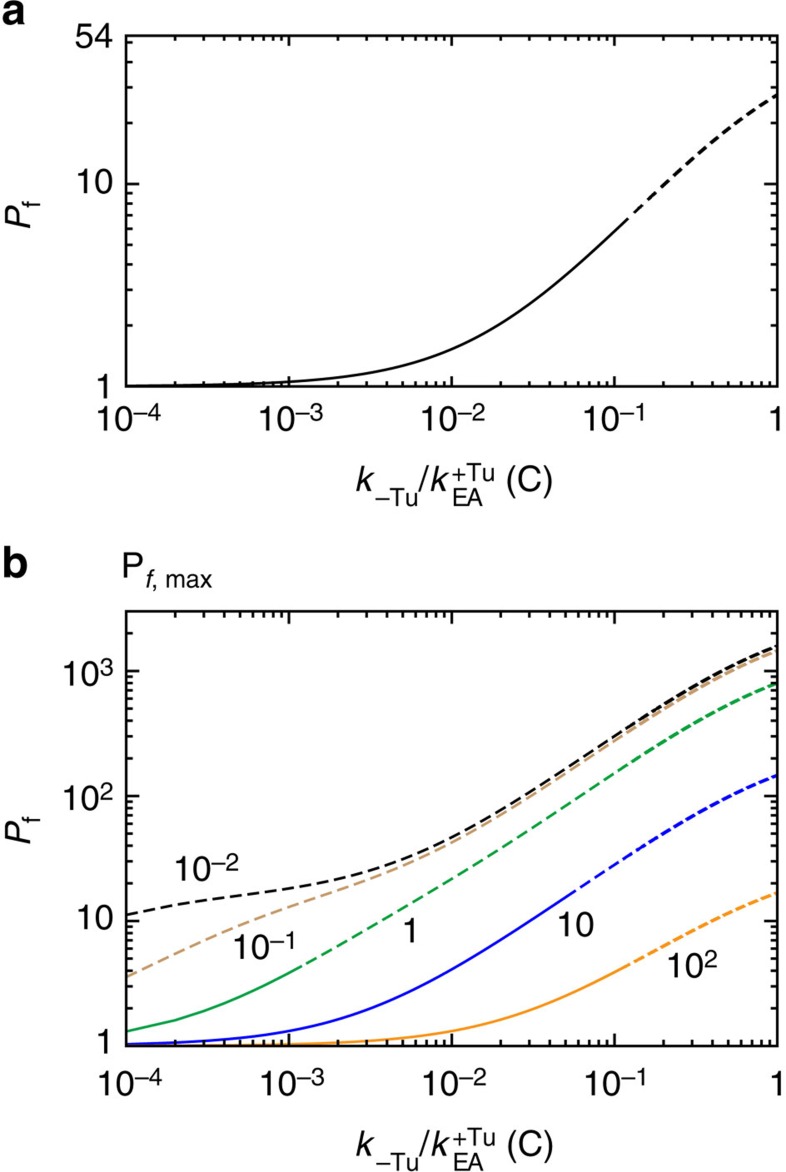
Rate of EF-Tu dissociation influences the proofreading factor *P*_f_. (**a**) *P*_f_ is shown as a function of 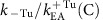 for the induced fit mechanism of proofreading (Fig. 4c). For this calculation, accommodation of cognate tRNA is modelled as being 50-fold faster than near-cognate: 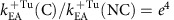. Only 
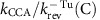
 =10 is shown since *P*_f_ is only sensitive to this ratio when it is <1. (**b**) For the EA-destabilized mode of proofreading (Fig. 4d), *P*_f_ depends on the relative rates of EF-Tu dissociation 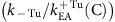 and 3′-CCA accommodation 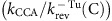. Each curve is labelled by the value of the relative rate of 3′-CCA accommodation (
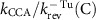
). A representative value of ΔΔ*F*_NC_=8 *k*_B_*T* was used to demonstrate the overall behaviour of *P*_f_. In contrast to the induced fit description, proofreading through EA destabilization depends on the value of *k*_CCA_. This is because, when tRNA is in the EA basin, the reverse step for near-cognate tRNA, with a rate of 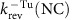, will be competitive with forward accommodation (rate of *k*_CCA_). In contrast, the reverse barrier is large for all molecules in the induced-fit framework. Common to both descriptions is that fidelity increases monotonically with the rate of EF-Tu dissociation *k*_−Tu_. Solid (dashed) lines distinguish between regions where efficiency is greater (less) than 0.9. As the rate of EF-Tu dissociation increases, the efficiency decreases. This suggests that EF-Tu dissociation should be sufficiently slow, such that it may accelerate elbow accommodation (increase efficiency), yet fast enough to allow for rejection (increase selectivity).

To explore the potential biological implications of EF-Tu-accelerated elbow accommodation, we used the extended kinetic model (Fig. 4b) to study an induced fit description of accommodation. Induced fit dynamics is consistent with bulk kinetic measurements that have found the rate of accommodation to be faster for cognate aa-tRNA, than for near-cognate^12^. These differences in rates may be schematically described as an increase in the free-energy barrier for near-cognate molecules (Fig. 4c). In terms of the kinetic model, we simply assigned the rate of elbow accommodation to be 50-fold slower for near-cognate molecules. We then calculated the proofreading factor and efficiency as functions of the EF-Tu dissociation rate *k*_−Tu_ (Fig. 5a).

For codon–anticodon mismatches that obey induced fit dynamics, the extended kinetic model predicts the proofreading factor will depend monotonically on *k*_−Tu_. When EF-Tu dissociation is very slow, *P*_f_ approaches 1 (no proofreading), since EF-Tu is assumed to impede aa-tRNA dissociation from the ribosome, irrespective of the codon. Accordingly, the efficiency would be high (Fig. 5a, solid line) since all molecules would eventually reach the accommodated state A. *P*_f_ then increases with *k*_−Tu_, indicating that kinetic partitioning of cognate and near-cognate molecules is possible^12^. As *k*_−Tu_ is increased, it also becomes increasingly likely that EF-Tu will dissociate before cognate elbow accommodation, which leads to population of the A/T^−Tu^ state. When this occurs, the barrier encountered by aa-tRNA is expected to be significantly increased in magnitude (Fig. 2b). Thus, the probability of rejecting a cognate aa-tRNA will increase, and the efficiency will decrease (Fig. 5a, dashed line). This behaviour suggests a very dynamic picture of proofreading, where EF-Tu dissociation should be sufficiently slow that it can assist accommodation (increase efficiency), yet fast enough to allow for rejection (enable selectivity).

In addition to increasing the barrier height, near-cognate interactions may also destabilize the A/T ensemble, which could lead to codon-dependent value of *k*_reject_. In support of this, near-cognate tRNAs are more rapidly rejected than cognate molecules^12^. Since this will amplify the degree of proofreading, the values of *P*_f_ described here should be considered lower bounds.

### Balancing EF-Tu dissociation and reverse accommodation

While the above analysis suggests that EF-Tu can play a pivotal role within an induced fit framework, we now address the relevance of EF-Tu-accelerated accommodation within an alternate kinetic description for proofreading. Specifically, we ask how EF-Tu-accelerated accommodation may contribute to proofreading if the EA ensemble were to be destabilized by near-cognate interactions. Recent structural models suggest that the steric characteristics of the decoding centre may lead to destabilization of the accommodated conformation for near-cognate aa-tRNA molecules^64^. That is, when aa-tRNA adopts the A/A conformation, the decoding centre (h18, h44, S12 and H69) sterically confines the first two bases, such that they are forced to adopt a cognate-like geometry. A near-cognate pair is then energetically penalized, relative to its relaxed state, by either breaking its hydrogen bonds or distorting the decoding centre. Free-energy calculations have estimated the penalty for a near-cognate codon to be ∼10–15 *k*_B_*T* (ref. 43), though it is not known how this is distributed between the A/T, transition state, EA and A/A ensembles. As discussed in the previous section, destabilization of the TSE (increasing the barrier height) would lead to induced fit dynamics and destabilization of the A/T ensemble would likely increase the rate of rejection *k*_reject_. To complement these landscape descriptions, we now considered the kinetic effects of EA destabilization (ΔΔ*F*_NC_ in Fig. 4d). If the EA ensemble were destabilized by near-cognate interactions, the reverse-elbow accommodation rearrangement could serve as a proofreading step, where codon-dependent destabilization would lead to tRNA-dependent rates of reverse elbow accommodation *k*_rev_.

If near-cognate interactions destabilize the EA ensemble, the proofreading factor *P*_f_ will depend monotonically on the rate of EF-Tu dissociation (Fig. 5b). Again, the physical interpretation for this dependence is that EF-Tu dissociation should be sufficiently fast that near-cognate molecules may be rejected, yet slow enough to maintain efficient accommodation of cognate tRNA. An interesting qualitative distinction between our analysis of the induced fit framework and the EA-destabilized framework is that in the latter, *P*_f_ and *E* depend on the rate of 3′-CCA end accommodation. This is due to the fact that the elbow accommodation barrier is predicted to be very small for all tRNA molecules when EF-Tu is present, in the EA-destabilized framework. Thus, EF-Tu would load cognate and near-cognate tRNA molecules into the marginally stable EA intermediate. Smaller values of *k*_CCA_ would be associated with a prolonged sampling of the EA^−Tu^ state, which would increase the probability reaching the A/T^−Tu^ state, for all tRNA molecules. By returning to the A/T^−Tu^ state, the tRNA may be subsequently rejected, which would reduce the chance of incorporating a mistake, while also reducing the efficiency.

The unique influence of *k*_CCA_ within the EA-destabilized proofreading approach provides an avenue for experimentally determining the extent to which this mechanism is employed. For example, it may be possible to introduce mutations near the PTC that specifically increase/decrease *k*_CCA_. A strong dependence of fidelity on these mutations would then be consistent with the utilization of a reverse-elbow proofreading mechanism.

## Discussion

It is widely recognized that proofreading by the ribosome is accomplished during a conformational step related to accommodation^1^,^4^,^12^,^13^,^15^,^17^,^18^,^19^,^20^,^21^,^22^,^23^,^24^,^25^,^27^,^28^,^30^,^32^,^33^,^63^. In an effort to provide insights into the physical–chemical properties of proofreading, we have analysed aa-tRNA elbow accommodation through the lens of energy landscape theory. Our simulations predict that EF-Tu can drive aa-tRNA towards a partially accommodated intermediate. By integrating this result within a kinetic model, we have provided two explanations for how EF-Tu may facilitate efficient proofreading. The most striking result is that a balance between the rates of EF-Tu dissociation and aa-tRNA accommodation can heavily influence the level and efficiency of proofreading.

From a physical perspective, the current study demonstrates how the structure of the ribosome can shape the free-energy landscape that governs biological dynamics. In the context of accommodation, multiple free-energy barriers are introduced by steric features that impede aa-tRNA motion. Here, we have shown how EF-Tu can help aa-tRNA overcome one such steric obstacle, H89. Relative to general discussions pertaining to the stochasticity of elongation, the predicted action of EF-Tu is reminiscent of the Brownian ratchet model of elongation^65^, where the steric contributions of EF-Tu increase the probability of tRNA crossing a large free-energy barrier.

Taken together, the presented simulations and kinetic model reveal how the structure of the ribosome can contribute to efficient proofreading. However, with the critical role of gene expression, the ribosome has likely evolved redundant strategies^19^,^44^ to ensure that accurate selection is robust across aa-tRNA species and cellular conditions. As biophysical investigations continue to explore the many facets of ribosome dynamics, the current study illustrates how the combination of experimental observations, computational tools and theoretical modelling may be used to identify robust biomolecular relationships that underly function.

## Methods

### Structure-based model (SBM)

All simulations employed a structure-based model (SBM)^47^. The SBM was implemented as in ref. 48, with the exception that non-specific electrostatic interactions are also included here. In the SBM, the potential energy minimum is defined by the A/A conformation^25^. All non-hydrogen atoms are included and each atom is represented as a bead of unit mass. Bond lengths, bond angles, improper dihedrals and planar dihedrals are maintained by harmonic potentials. The dihedral interactions are defined such that each angle is at a minimum in the A/A conformation. Non-bonded atom pairs that are in contact in the A/A conformation are given attractive interactions. Since intermolecular arm and 3′-CCA contacts only form after elbow accommodation^35^,^36^, they were not included in the current model^48^. This modification was introduced so that the calculations could specifically focus on the elbow accommodation step, and not the subsequent rearrangements. Additionally, stabilizing interactions between the A-site tRNA and P-site tRNA (elbow contacts) were scaled by 0.5. This reduction in the strength of A/A-specific contacts is in accordance with each contact representing effective interatomic energetics^49^. That is, the intramolecular interactions that maintain the structural integrity of the ribosome and each tRNA molecule are effectively more stable than the transiently formed intermolecular interactions between tRNA molecules. All atom pairs that do not interact via bonded or contact terms were given repulsive interactions, which account for the atomic excluded volume. In the current implementation of the SBM, non-specific electrostatic interactions between aa-tRNA and the ribosome were also included. The functional form of the potential is given by:


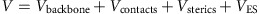



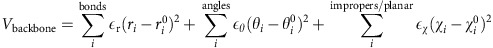



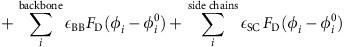



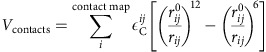



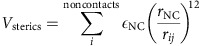



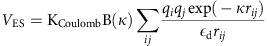



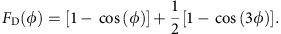


*V*_contacts_ describes short-range attractive interactions between atoms *i* and *j* that are in contact in the A/A conformation. A 4 Å cutoff was used to define the native contacts^66^. As described elsewhere^67^, three ratios are defined to assign values of 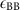, 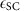 and 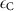. (1) 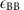 and 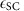 are scaled so that 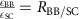. (2) The energetic weight of each dihedral and contact is also scaled, such that the ratio of total contact energy to total dihedral energy 
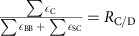
, is satisfied. (3) The total stabilizing energy is set, such that 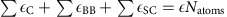, where 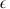 is the reduced energy unit. *R*_BB/SC_=2 for protein, *R*_BB/SC_=1 for RNA and *R*_C/D_=2. 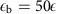, 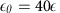, 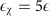 for improper dihedrals, 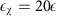 for planar dihedrals, and 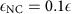. 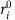, 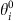, 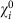, 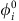 and 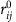 are given the values found in the A/A crystal structure, and *r*_NC_=2.5 Å. Contacts between stacked RNA bases (bases adjacent in sequence) are scaled by 1/3 relative to all other contacts. Electrostatic interactions were modelled by a Debye-Hückel potential (*V*_ES_), as in ref. 50. According to Debye–Hückel theory, the electrostatic interactions are screened by monovalent ions with a characteristic Debye screening length *κ*^−1^. For dilute solutions of monovalent ions at room temperature, *B*(*κ*)≈1 and 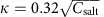. Here, an effective salt concentration of 0.05 M was used, corresponding to *κ*=1.4 nm, and a dielectric 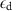 of 80 rescaled into reduced units (see Supplementary Methods). Crystallographic magnesium ions were given a charge +2, the phosphorous atom of each nucleic acid was given a charge of −1, and Arg, Lys, Asp and Glu residues were charged ±1 (His were uncharged). 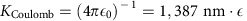.

### Structural models

A crystallographic model of the A/A structure (PDB code: 3I8F)^25^ was used to define the EF-Tu-free (−Tu) SBM. To reduce the computational requirement of each simulation, only the atoms within a rectangular prism containing the accommodation corridor (23,888 of ∼150,000 atoms) were explicitly included in the simulations. This reduction in the number of atoms resulted in numerous boundary atoms having interactions removed from the model. To avoid introducing artifacts, the boundary atoms were subject to harmonic restraints centred at the crystallographic coordinates. The strength of each restraint was determined through an iterative fluctuation-matching protocol, as described previously^48^. There is excellent agreement between the fluctuations in the truncated system and the full ribosome, where the correlation coefficient of the atomic root mean squared fluctuations (RMSF) values is 0.98 (Supplementary Fig. 4).

The structural model containing EF-Tu in its GTP conformation (Tu^GTP^) was generated by aligning an A/T structure of the ribosome with EF-Tu bound^29^ to the small ribosomal subunit of the −Tu model. The aligned coordinates of EF-Tu were then directly used to restrain EF-Tu during the simulations. The alignment process introduced no steric clashes between EF-Tu and the ribosomal corridor, where no atoms were closer than 3 Å, and 20 atom pairs were within 4 Å. The two models of possible post-Pi-release conformations of EF-Tu, 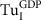 and 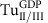, were generated by fitting the GDP crystal structure of EF-Tu (ref. 59) to either domain I or the II/III superdomain of Tu^GTP^. For both, the RMSD of the fitted atoms was <3 Å, showing that the conformational change in EF-Tu can be predominantly described as a relative displacement of domains.

### Molecular dynamics simulations

Free-energy profiles were calculated from molecular dynamics simulations performed with GROMACS v4.5.3 (ref. 68). The input forcefield files were generated by the SMOG web server (http://smog-server.org)^67^. In simulations that included EF-Tu, each atom in EF-Tu was restrained to the assigned position. Umbrella sampling was applied by imposing restraints on the distance between the geometric centres of the tRNA elbows. About the TSE, an additional umbrella restraint roughly perpendicular to the elbow displacement was necessary to sample both the major and minor groove routes (see Supplementary Fig. 5 for details). The Weighted Histogram Analysis Method^69^ was used to obtain the free energy as a function of *R*_elbow_ (distance between U8 in the P-site tRNA and U60 in the A-site tRNA, as defined as in ref. 48). The positions of individual umbrellas were sufficiently close that the full range of *R*_elbow_ values were sampled (Supplementary Fig. 6). Umbrella simulations were performed twice, once after initializing the simulations by iteratively increasing *R*_elbow_ and then again by decreasing *R*_elbow_ (Supplementary Fig. 7). For a summary of all systems simulates, see Supplementary Table 1. See Detailed Methods in Supplementary Information for full simulation details.

### Data availability

The data that support the findings of this study are available from the corresponding author upon reasonable request.

## Additional information

**How to cite this article:** Noel, J. K. & Whitford, P. C. How EF-Tu can contribute to efficient proofreading of aa-tRNA by the ribosome. *Nat. Commun.* **7,** 13314 doi: 10.1038/ncomms13314 (2016).

**Publisher's note:** Springer Nature remains neutral with regard to jurisdictional claims in published maps and institutional affiliations.

## Supplementary Material

Supplementary Information Supplementary Figures 1-7, Supplementary Table, Supplementary Methods and Supplementary References.
