# Supplementary material for: How EF-Tu can contribute to efficient proofreading of aa-tRNA by the ribosome
Source: Nat Commun. 2016 Oct 31;7:13314. doi: 10.1038/ncomms13314 (PMC5095583; doi:10.1038/ncomms13314)
Supplement: Supplementary Information — Supplementary Figures 1-7, Supplementary Table, Supplementary Methods and Supplementary References. [file ncomms13314-s1.pdf]

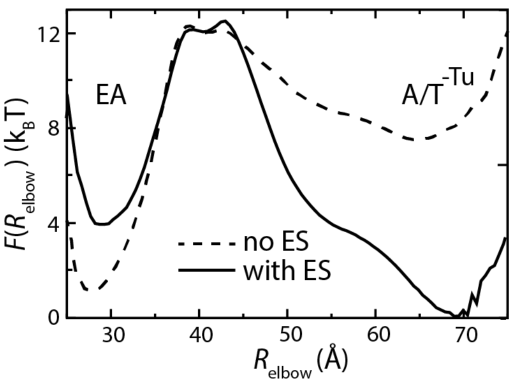

SUPPLEMENTARY FIG. 1. Position of the transition state ensemble (TSE) along R_{elbow} is not affected by non-specific electrostatic interactions between the aa-tRNA and H89. Including electrostatics in the model does not shift the location of the elbow accommodation barrier, which was tested by comparing the models Tu-free (non-specific electrostatics included, solid line, labeled "with ES") and noES-free (electrostatics-free, dashed line, labeled "no ES"). While electrostatics do not shift the location of the barrier, including electrostatics does shift the stability in favor of the $\mathrm{A/T}^{-\mathrm{Tu}}$ basin due to the density of repulsive RNA-RNA interactions in the EA ensemble. Additionally, when electrostatics is included, the tRNA is less confined in the $\mathrm{A/T}^{-\mathrm{Tu}}$ basin and can be positioned further from the ribosomal RNA. This movement away from the accommodation corridor is described by a shift in the position of the free energy minimum of the $\mathrm{A/T}^{-\mathrm{Tu}}$ basin (R_{elbow} shifts from 65 Å to 69 Å). The location of the EA basin along R_{elbow} is negligibly altered by electrostatics.

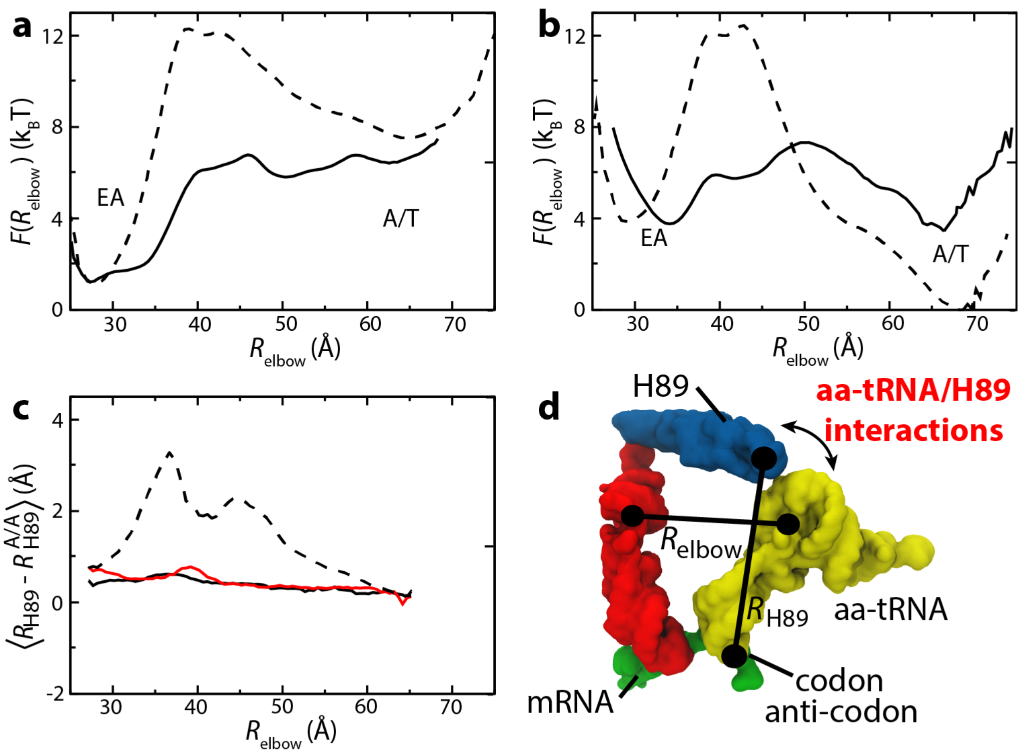

SUPPLEMENTARY FIG. 2. Steric interactions with Helix 89 lead to a free-energy barrier. Free energy profiles are shown as functions of the elbow coordinate R_{elbow}, with aa-tRNA/H89 interactions excluded, or included, from the model. Free energies are shown for (**a**) the noH89-noES-free (solid) and noES (dashed) models, and (**b**) the noH89-free (solid) and Tu-free (dashed) models. Removal of aa-tRNA/H89 interactions (i.e. a "virtual mutation" [1]) dramatically reduces the barrier to accommodation. This directly shows that H89 imposes the barrier obtained with the structure-based model. (**c**) Comparison of the displacement of H89 relative to its crystallographic position in the Tu-free (dashed), noH89-noES-free (black), and noH89-free (red) models. (**d**) Structural representation of coordinates used in (**a-c**).

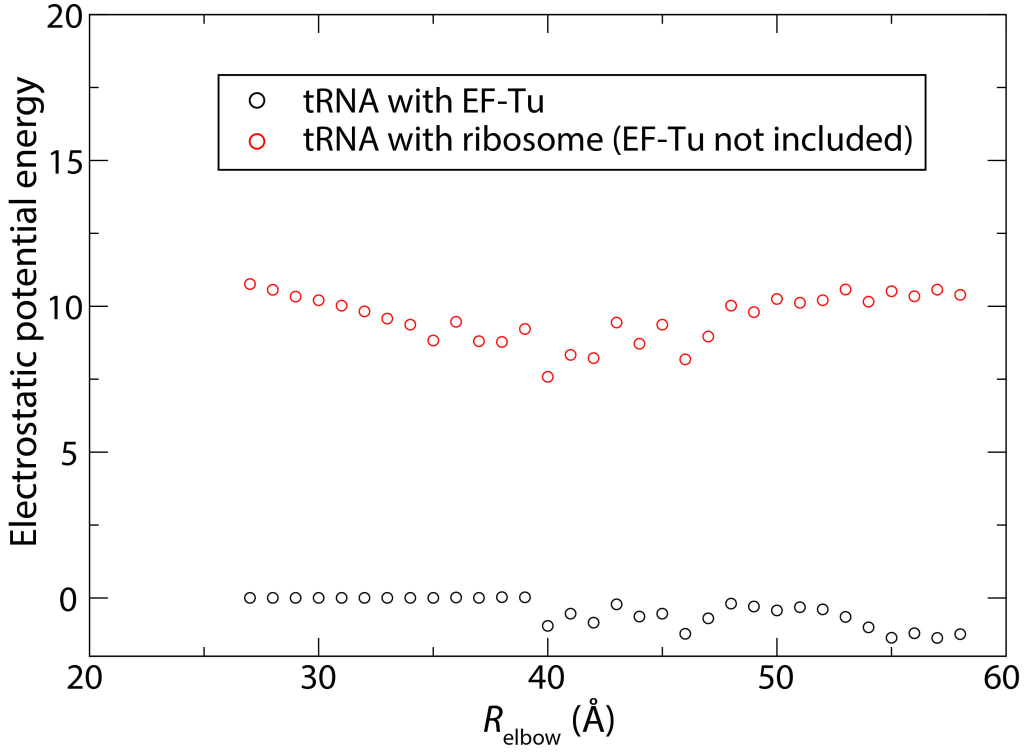

SUPPLEMENTARY FIG. 3. Electrostatic interactions between aa-tRNA/EF-Tu stabilize the A/T ensemble, relative to the EA ensemble. As described in the main text, EF-Tu bound to the ribosome in its A/T orientation dramatically destabilizes the A/T basin. To determine whether this destabilization is caused by electrostatic interactions between aa-tRNA and EF-Tu, the electrostatic potential between aa-tRNA and EF-Tu was calculated as a function of elbow distance (black circles). The aa-tRNA-EF-Tu interactions have a lower potential energy in the A/T ensemble (larger values of R_{elbow}) than the EA ensemble, which confirms that the observed destabilization of the A/T ensemble is due to steric interactions, and not electrostatic repulsion. Similarly, the electrostatic potential between aa-tRNA and non-EF-Tu atoms (red circles) also increases as the tRNA accommodates.

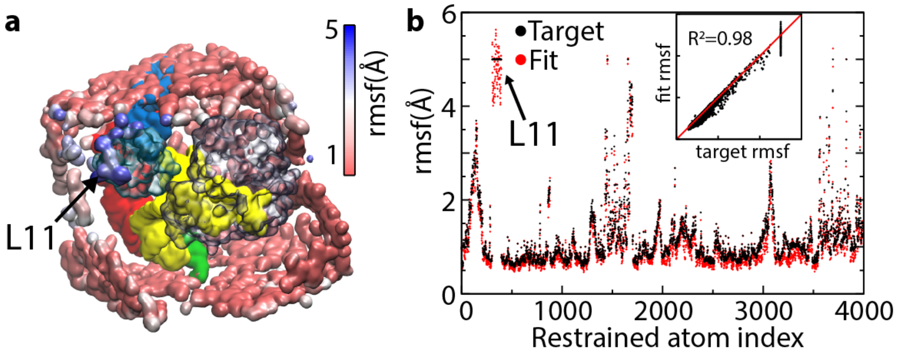

SUPPLEMENTARY FIG. 4. Harmonic restraints are applied to atoms on the boundary of the simulation box. (**a**) The target atomic rmsf values (i.e. values obtained from simulations of a full ribosome) are shown for each boundary atom. A boundary atom is defined as an atom that has a native interaction (bond, bond angle, contact) with an atom that is removed at the truncation step. (**b**) Value of the target rmsf values, compared to those obtained for the truncated system after fluctuation matching was employed [2]. The target value for each atom was capped at 5 Å since this is roughly the limit for determining coordinates in an x-ray crystal structure.

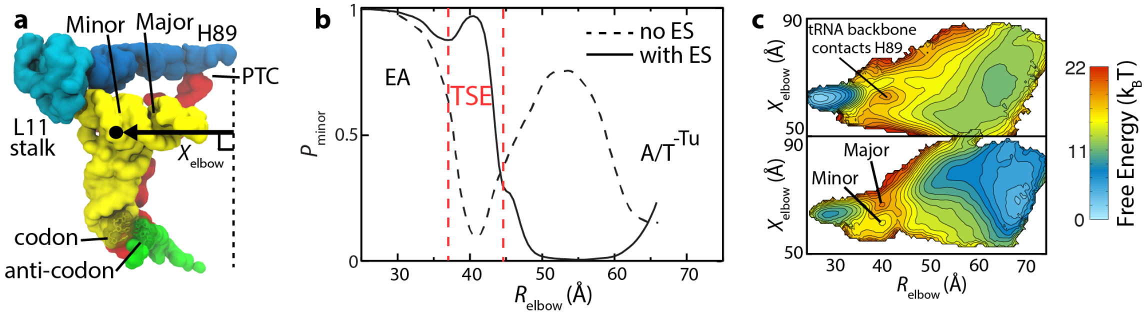

SUPPLEMENTARY FIG. 5. H89-tRNA interactions in the TSE. (**a**) aa-tRNA can pass by H89 through either the major or minor groove, and electrostatic interactions favor the minor groove pathway. This subtle structural heterogeneity in the transition state ensemble is roughly perpendicular to R_{elbow} and is described by X_{elbow}. (**b**) The switch between major/minor groove can be quantified by P_{minor}, the probability that the minimum distance from the minor groove to H89 is smaller than the minimum distance from the major groove to H89. In the TSE region (highlighted by red dashed lines), non-specific electrostatic interactions favor the minor groove (solid line, "with ES") and the electrostatics-free model favors the major groove (dashed line, "no ES"). This switch occurs because, in the absence of electrostatic repulsion between the phosphates of H89 and tRNA, it is sterically favorable to bypass H89 through the wider major groove. However, upon addition of non-specific electrostatics, H89 prefers the more sterically restricted minor groove over the high density of repulsive phosphates in the major groove (**c**). The multiple routes highlight the flexibility of the tRNA molecule and show how modulating the strength of electrostatics, e.g. changing ionic concentrations, can affect minor mechanistic aspects of accommodation.

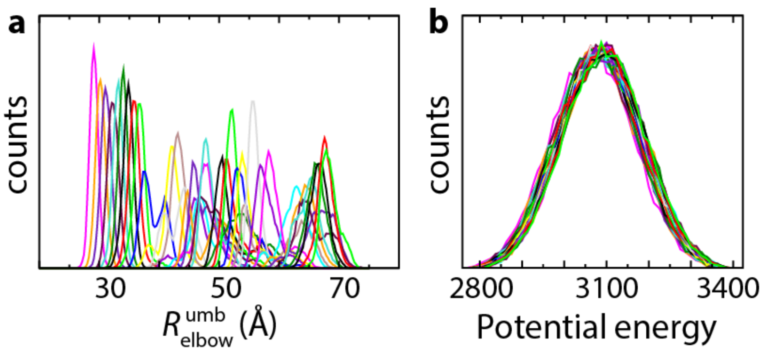

SUPPLEMENTARY FIG. 6. Sampled configuration space in umbrella sampling calculations. (**a**) Histograms of the umbrella coordinate $R_{\mathrm{elbow}}^{\mathrm{umb}}$ for 39 simulations with varying positions of the umbrella (i.e $R_{\mathrm{elbow}}^{\mathrm{umb0}}$) shows that the simulations sample the full range of values of the umbrella coordinate. (**b**) Potential energy histograms of the 39 umbrella simulations are all very similar.

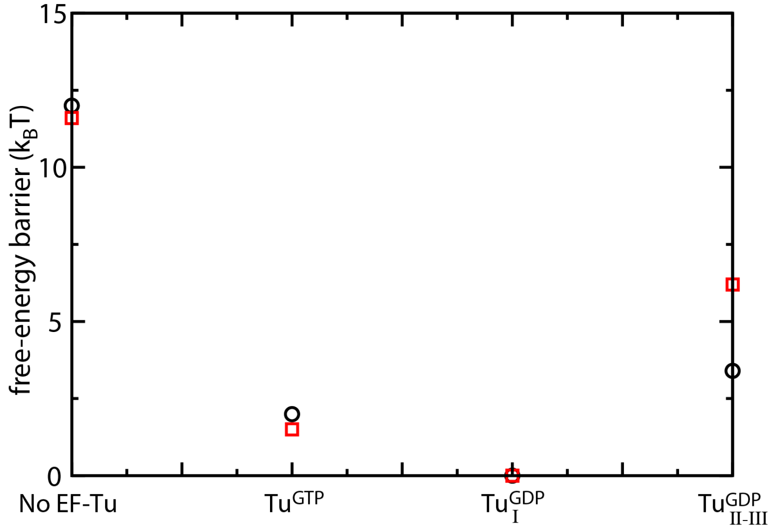

SUPPLEMENTARY FIG. 7. Free-energy barrier heights associated with forward elbow accommodation for each construct, obtained after equilibration in the EA-to-A/T direction (circles) and the A/T-to-EA direction (squares). The ordering of barrier heights and the large difference between the EF-Tu-free and EF-Tu-present barriers are robust to equilibration protocol.

SUPPLEMENTARY TABLE 1. Summary of models

No.	Model Name	Description
1	Tu-free (-Tu)	Non-specific electrostatics between aa-tRNA and accommodation corridor[a]
2	$\mathrm{Tu}^{\mathrm{GTP}}$	Tu-free model with canonical EF-Tu·GTP[b]
3	$\mathrm{Tu}^{\mathrm{GDP}}_{\mathrm{I}}$	Tu-free model with EF-Tu·GDP[c] fit via N-terminal domain[d]
4	$\mathrm{Tu}^{\mathrm{GDP}}_{\mathrm{II/III}}$	Tu-free model with EF-Tu·GDP fit via C-terminal domains
5	noES	Baseline (no electrostatics) structure-based model of Tu-free
6	noH89-noES-free	noES with no interaction between aa-tRNA and H89
7	noH89-free	Tu-free with no interaction between aa-tRNA and H89

[a] Structure-based model created from an A/A structure of the ribosome [3].

[b] EF-Tu from an A/T crystal structure [4].

[c] EF-Tu from crystal structure of isolated EF-Tu in complex with GDP [5].

[d] Orientation of EF-Tu·GDP bound to the ribosome obtained by aligning the N-terminal domain to EF-Tu·GTP.

SUPPLEMENTARY METHODS

Simulation details

Structure-based simulations were performed using the GROMACS v4.5.3 software package [6]. The input forcefield files were generated by the SMOG web server v1.2.2 (http://smog-server.org) [7]. Each simulation was typically performed on 32 cores. The time step τ was 0.002. Temperature was controlled through Langevin dynamics with a coupling constant of 1. The simulation temperature was determined by matching the atomic RMS fluctuations from simulation to experimental B-factors [8] according to Ref. [9]. This implicated the temperature range 50-60 in GROMACS units or 0.42-0.5 $\epsilon/\mathrm{k_B}$ in reduced units. The simulations were run at 60, but similar results are obtained at 50 (see [2]). The strength of the electrostatic term was scaled to its corresponding value in reduced units by matching its energy scale with the temperature of the system [10]. Compared to the experimental temperature ($\mathrm{k_B}\times$300 K)/[1 kJ mol^{-1}] = 2.5, the simulations are in the range $0.42 < \mathrm{k_B T}/\epsilon < 0.5$, so the "reduced" dielectric of water ϵ_d (normally 80) should be scaled to between 80×2.5/0.5 and 80×2.5/0.42, *i.e.* 400 to 480. Since the screening of non-crystallographic magnesium is not accounted for in our Debye-Hückel treatment, we chose the lower range of electrostatic strength to compensate, $\epsilon_\mathrm{d} = 480$. The van der Waals and electrostatic interaction cutoff distance was set to 1.5 nm, with a forth-order polynomial switching function being introduced at 1.2 nm for electrostatics. Lookup table generations scripts are available as part of the SMOG 2 distribution [7].

The reaction coordinates for umbrella sampling were: 1) the elbow distance $R^{\mathrm{umb}}_{\mathrm{elbow}}$ determined by the distance between the geometric centers of 549 atoms in the elbows of the P-site and A-site tRNA, which is highly correlated with R_{elbow} and 2) X_{elbow} (Supplementary Fig. 5). Harmonic umbrellas of the form $V_{R,\mathrm{elbow}} = 150\epsilon(R^{\mathrm{umb}}_{\mathrm{elbow}} - R^{\mathrm{umb0}}_{\mathrm{elbow}})^2$ and were used with $R^{\mathrm{umb0}}_{\mathrm{elbow}} = 2.7 + n0.1$ nm (Supplementary Fig. 6). Similarly, $V_{X,\mathrm{elbow}} = 4\epsilon(X^{\mathrm{umb}}_{\mathrm{elbow}} - X^{0}_{\mathrm{elbow}})^2$ with $X^{0}_{\mathrm{elbow}} = (5, 6, 7, 8)$. Near EA $X^{0}_{\mathrm{elbow}} = 6$, near the barrier $X^{0}_{\mathrm{elbow}} = (5, 6, 7, 8)$, and near A/T $X^{0}_{\mathrm{elbow}} = (6, 7, 8)$. The main function of the X_{elbow} umbrella was to enforce sampling of both the major and minor groove routes (Supplementary Fig. 5). X^{0}_{elbow} is the distance from the plane defined by three atoms on the interior edge of the box: P atom of C2619 in the large subunit, P atom of C55 of the small subunit and P atom of G710 of small subunit. In the simulation, this plane was aligned with the y-z plane, which allowed the X_{elbow} umbrella to be

implemented by a GROMACS position restraint applied along the x-direction. The Weighted Histogram Analysis Method [11] was used to combine data from the umbrella simulations into a single free-energy profile. Each simulation was performed for 4×10^7 time steps. To verify convergence, the profiles were re-calculated using the first and second half of each data set.

Equilibration of the umbrella calculations was initiated from a randomly selected EA configuration that was obtained in an earlier trajectory [2]. The set of initial configurations for the umbrella simulations was generated by iteratively changing $R^{\mathrm{umb0}}_{\mathrm{elbow}}$ from 2.7 nm to 6.5 nm in steps of 0.1 nm every 5×10^5 steps. The last frame of each $R^{\mathrm{umb0}}_{\mathrm{elbow}}$ run was used as the initial structure of the umbrella centered at $R^{\mathrm{umb0}}_{\mathrm{elbow}}$. This was performed initially without the use of the X_{elbow} umbrella. To equilibrate systems for different values of X^0_{elbow}, the same protocol was followed individually for each value of $R^{\mathrm{umb0}}_{\mathrm{elbow}}$, starting with the value of X^0_{elbow} that was closest to the initial configuration being equilibrated.

To test for possible hysteresis effects that can arise from the choice of equilibration protocol, we repeated the umbrella sampling simulations for the EF-Tu-free system, as well as for all three EF-Tu-present systems. For these additional calculations, an A/T configuration was randomly selected for each system, where R_{elbow} and R_{CCA} adopt extended A/T-like values. Starting from these extended configurations, each system was simulated for 5×10^5 steps with $X^0_{\mathrm{elbow}} = 6$ and $R^{\mathrm{umb0}}_{\mathrm{elbow}}$ set to 6.5 (EF-Tu free), or 6.0 (with EF-Tu). For each system, $R^{\mathrm{umb0}}_{\mathrm{elbow}}$ was iteratively decreased by 0.1nm and simulated for 5×10^5 steps. After equilibration of each run, $R^{\mathrm{umb0}}_{\mathrm{elbow}}$ was iteratively incremented by 1nm, which was followed by simulation of 5×10^5 steps. Using these equilibrated systems, production simulations were performed for 4×10^7 steps, from which free-energy profiles were calculated. In comparison with the profiles described in the main text, the height of the free-energy barrier for forward elbow accommodation shows negligible dependence on equilibration protocol (Supplementary Fig. 7). The most significant change is an increase in barrier height for the $\mathrm{Tu}^{\mathrm{GDP}}_{\mathrm{II-III}}$ construct, by $\approx 3\mathrm{k_B T}$. However, the ordering of barrier heights is consistent. Further, the large decrease in barrier ($6\mathrm{k_B T}$, or greater) when EF-Tu is present, relative to when it is absent, is observed in both sets of simulations. Accordingly, the observation of EF-Tu-accelerated elbow accommodation is robust across all calculations.

Calculating rates from free energy profiles

Since R_{elbow} is an accurate indicator of when the aa-tRNA is on a transition path, and movement about the TSE is diffusive along this coordinate [2], we use it to describe the kinetics of accommodation. With these criteria satisfied, the probability density $P(\rho, t)$ of a system along a reaction coordinate ρ may be described by the Fokker-Planck equation

$$\frac{\partial}{\partial t}P(\rho,t) = \frac{\partial}{\partial \rho}\left[D(\rho)\left(\frac{\partial P(\rho,t)}{\partial \rho} + P(\rho,t)\frac{\partial \beta F(\rho)}{\partial \rho}\right)\right] \tag{1}$$

where $D(\rho)$ is the diffusion coefficient. The method of first passage times provides a way to compute rates of movement along the free energy surface $F(\rho)$ as described in [12]. The rate k, or first passage time τ, may be expressed in terms of the free energy according to:

$$1/k = \langle\tau\rangle = \int_{\rho_{\text{A/T}}}^{\rho_{\text{EA}}} d\rho \int_{\infty}^{\rho} d\rho' \frac{\exp[F(\rho) - F(\rho'))/k_{\text{B}}T]}{D(\rho')}. \tag{2}$$

We use the value of $D(R_{\text{elbow}}) = 110$ Å^2/s, as obtained in previous explicit-solvent simulations [13]. Note that since the previous calculations were performed for the slightly different elbow distance coordinate (distance between P-tRNA U8 and aa-tRNA U47), there is a small degree of uncertainty in this value. Assuming the diffusion coefficient is roughly position independent, the dominant contribution to the rate is the height and shape of the free energy barrier. To obtain the rates, we numerically evaluate the integral (*i.e.* replacing ρ for R_{elbow}) for each landscape. These rates were then used as input to the kinetic model, as described in the next section. k_{CCA} and $k_{-\text{Tu}}$ are not calculated in this study and are treated as parameters defining a range of possible proofreading efficiencies and selectivities.

Details of the extended kinetic model

The extended kinetic model describes conformational events that occur after initial selection, which means the input concentration to the kinetic flow [A] is already heavily biased towards cognate pairs. The rates of rejection and accommodation can be estimated for an ensemble of ribosomes with a steady-state approximation of the kinetic scheme in Fig. 4 in the main text. Three equations for the intermediate states at steady-state are given by:

$$\frac{d[\mathrm{EA}^{+\mathrm{Tu}}]}{dt} = 0 = -(k_{\mathrm{rev}}^{+\mathrm{Tu}} + k_{-\mathrm{Tu}} + k_{\mathrm{CCA}})[\mathrm{EA}^{+\mathrm{Tu}}] + k_{\mathrm{EA}}^{+\mathrm{Tu}}[\mathrm{A/T}^{+\mathrm{Tu}}] \rightarrow [\mathrm{EA}^{+\mathrm{Tu}}] = \frac{k_{\mathrm{EA}}^{+\mathrm{Tu}}}{k_{\mathrm{rev}}^{+\mathrm{Tu}} + k_{-\mathrm{Tu}} + k_{\mathrm{CCA}}} \tag{3}$$

$$\frac{d[\mathrm{EA}^{-\mathrm{Tu}}]}{dt} = 0 = -(k_{\mathrm{rev}}^{-\mathrm{Tu}} + k_{\mathrm{CCA}})[\mathrm{EA}^{-\mathrm{Tu}}] + k_{-\mathrm{Tu}}[\mathrm{EA}^{-\mathrm{Tu}}] \rightarrow [\mathrm{EA}^{-\mathrm{Tu}}] = \frac{k_{-\mathrm{Tu}}}{k_{\mathrm{rev}}^{-\mathrm{Tu}} + k_{\mathrm{CCA}}} \tag{4}$$

$$\frac{d[\mathrm{A/T}^{+\mathrm{Tu}}]}{dt} = 0 = -(k_{\mathrm{EA}}^{+\mathrm{Tu}} + k_{-\mathrm{Tu}})[\mathrm{A/T}^{+\mathrm{Tu}}] + k_{\mathrm{rev}}^{+\mathrm{Tu}}[\mathrm{EA}^{+\mathrm{Tu}}] + k_{\mathrm{IS}}[\mathrm{Ter} + \mathrm{Ribo}] \tag{5}$$

where $[\mathrm{A/T}^{+\mathrm{Tu}}]$ is the state immediately after GTP hydrolysis and phosphate release and $[\mathrm{EA}^{+/-\mathrm{Tu}}]$ are the elbow-accommodated states with and without EF-Tu. Two equations for each of the products are given by:

$$\frac{d[\mathrm{A}]}{dt} = k_{\mathrm{CCA}}[\mathrm{EA}^{+\mathrm{Tu}}] + k_{\mathrm{CCA}}[\mathrm{EA}^{-\mathrm{Tu}}] \tag{6}$$

$$\frac{d[\mathrm{A/T}^{-\mathrm{Tu}}]}{dt} = k_{\mathrm{rev}}^{-\mathrm{Tu}}[\mathrm{EA}^{-\mathrm{Tu}}] + k_{-\mathrm{Tu}}[\mathrm{A/T}^{+\mathrm{Tu}}] \tag{7}$$

where $[\mathrm{A/T}^{-\mathrm{Tu}}]$ is the extended basin for tRNA without EF-Tu. With two equations and two unknowns ($[\mathrm{EA}^{+\mathrm{Tu}}]$ and $[\mathrm{EA}^{-\mathrm{Tu}}]$) one may find expressions for the rate to accommodate ($\frac{d[\mathrm{A}]}{dt}$) and reject ($\frac{d[\mathrm{A/T}^{-\mathrm{Tu}}]}{dt}$).

$$\frac{d[\mathrm{A}]}{dt} = \frac{k_{\mathrm{IS}}k_{\mathrm{CCA}}\left(k_{\mathrm{EA}}^{+\mathrm{Tu}}k_{\mathrm{EA}}^{-\mathrm{Tu}}(k_{\mathrm{CCA}} + k_{-\mathrm{Tu}}) + k_{\mathrm{EA}}^{+\mathrm{Tu}}k_{\mathrm{reject}}(k_{\mathrm{CCA}} + k_{-\mathrm{Tu}} + k_{\mathrm{rev}}^{-\mathrm{Tu}}) + k_{\mathrm{EA}}^{-\mathrm{Tu}}k_{-\mathrm{Tu}}(k_{\mathrm{CCA}} + k_{-\mathrm{Tu}} + k_{\mathrm{rev}}^{+\mathrm{Tu}})\right)}{\left(k_{\mathrm{rev}}^{-\mathrm{Tu}}k_{\mathrm{reject}} + k_{\mathrm{CCA}}(k_{\mathrm{reject}} + k_{\mathrm{EA}}^{-\mathrm{Tu}})\right)\left(k_{\mathrm{EA}}^{+\mathrm{Tu}}(k_{\mathrm{CCA}} + k_{-\mathrm{Tu}}) + k_{-\mathrm{Tu}}(k_{\mathrm{CCA}} + k_{-\mathrm{Tu}} + k_{\mathrm{rev}}^{+\mathrm{Tu}})\right)}[\mathrm{T} + \mathrm{R}] \tag{8}$$

$$\frac{d[\mathrm{A/T}^{-\mathrm{Tu}}]}{dt} = \frac{k_{\mathrm{IS}}k_{\mathrm{reject}}\left((k_{\mathrm{EA}}^{+\mathrm{Tu}}k_{-\mathrm{Tu}}k_{\mathrm{rev}}^{-\mathrm{Tu}}) + k_{-\mathrm{Tu}}(k_{\mathrm{CCA}} + k_{-\mathrm{Tu}} + k_{\mathrm{rev}}^{+\mathrm{Tu}})(k_{\mathrm{CCA}} + k_{\mathrm{rev}}^{-\mathrm{Tu}})\right)}{\left(k_{\mathrm{rev}}^{-\mathrm{Tu}}k_{\mathrm{reject}} + k_{\mathrm{CCA}}(k_{\mathrm{reject}} + k_{\mathrm{EA}}^{-\mathrm{Tu}})\right)\left(k_{\mathrm{EA}}^{+\mathrm{Tu}}(k_{\mathrm{CCA}} + k_{-\mathrm{Tu}}) + k_{-\mathrm{Tu}}(k_{\mathrm{CCA}} + k_{-\mathrm{Tu}} + k_{\mathrm{rev}}^{+\mathrm{Tu}})\right)}[\mathrm{T} + \mathrm{R}] \tag{9}$$

A reasonable approximation that helps simplify the expressions is $k_{\text{reject}} \gg k_{\text{EA}}^{-\text{Tu}}$. This gives,

$$\frac{d[\text{A}]}{dt} = \frac{k_{\text{IS}} k_{\text{CCA}} k_{\text{EA}}^{+\text{Tu}} (k_{\text{CCA}} + k_{-\text{Tu}} + k_{\text{rev}}^{-\text{Tu}})}{(k_{\text{rev}}^{-\text{Tu}} + k_{\text{CCA}}) \left(k_{\text{EA}}^{+\text{Tu}} (k_{\text{CCA}} + k_{-\text{Tu}}) + k_{-\text{Tu}} (k_{\text{CCA}} + k_{-\text{Tu}} + k_{\text{rev}}^{+\text{Tu}})\right)} [\text{T} + \text{R}] \quad (10)$$

$$\frac{d[\text{A}/\text{T}^{-\text{Tu}}]}{dt} = \frac{k_{\text{IS}} \left((k_{\text{EA}}^{+\text{Tu}} k_{-\text{Tu}} k_{\text{rev}}^{-\text{Tu}}) + k_{-\text{Tu}} (k_{\text{CCA}} + k_{-\text{Tu}} + k_{\text{rev}}^{+\text{Tu}})(k_{\text{CCA}} + k_{\text{rev}}^{-\text{Tu}})\right)}{(k_{\text{rev}}^{-\text{Tu}} + k_{\text{CCA}}) \left(k_{\text{EA}}^{+\text{Tu}} (k_{\text{CCA}} + k_{-\text{Tu}}) + k_{-\text{Tu}} (k_{\text{CCA}} + k_{-\text{Tu}} + k_{\text{rev}}^{+\text{Tu}})\right)} [\text{T} + \text{R}] \quad (11)$$

(12)

The selection ratio of proofreading P_{f} can be defined as the ratio of cognate $[\text{A}]^{\text{C}}$ to near/non-cognate $[\text{A}]^{\text{NC}}$ tRNA that arrive at the fully accommodated state. The rates that change based on the codon-anticodon favorability are the reverse rate $k_{\text{rev}}^{+/-\text{Tu}}(\mathbf{NC})$ for the EA basin destabilized mode of proofreading, and the forward rate $k_{\text{EA}}^{+/-\text{Tu}}(\mathbf{NC})$ for the induced-fit mode of proofreading. The selectivity for the induced-fit mode, P_{f}^{IF}, is given by:

$$P_{\text{f}}^{\text{IF}} = \frac{k_{\text{EA}}^{+\text{Tu}}(\mathbf{C})}{k_{\text{EA}}^{+\text{Tu}}(\mathbf{NC})} \frac{k_{\text{EA}}^{+\text{Tu}}(\mathbf{NC})(k_{\text{CCA}} + k_{-\text{Tu}}) + k_{-\text{Tu}}(k_{\text{CCA}} + k_{-\text{Tu}} + k_{\text{rev}}^{+\text{Tu}})}{k_{\text{EA}}^{+\text{Tu}}(\mathbf{C})(k_{\text{CCA}} + k_{-\text{Tu}}) + k_{-\text{Tu}}(k_{\text{CCA}} + k_{-\text{Tu}} + k_{\text{rev}}^{+\text{Tu}})}. \quad (13)$$

The expression is relatively simple and has basically no k_{CCA} dependence for $S \gg 1$. This is because k_{CCA} only plays a role if it is slow compared to $k_{\text{rev}}^{-\text{Tu}}(\mathbf{NC})$ and similar or larger than $k_{-\text{Tu}}$. For $S \gg 1$, $k_{-\text{Tu}}$ should be comparable to $k_{\text{EA}}^{+\text{Tu}}(\mathbf{C})$, i.e. fast.

The selectivity for the EA-destabilized description, P_{f}^{EA}, is given by:

$$P_{\text{f}}^{\text{EA}} = \frac{k_{\text{CCA}} + k_{-\text{Tu}} + k_{\text{rev}}^{-\text{Tu}}(\mathbf{C})}{k_{\text{CCA}} + k_{-\text{Tu}} + k_{\text{rev}}^{-\text{Tu}}(\mathbf{NC})} \frac{\left(k_{\text{rev}}^{-\text{Tu}}(\mathbf{NC}) + k_{\text{CCA}}\right) \left(k_{\text{EA}}^{+\text{Tu}}(k_{\text{CCA}} + k_{-\text{Tu}}) + k_{-\text{Tu}}(k_{\text{CCA}} + k_{-\text{Tu}} + k_{\text{rev}}^{+\text{Tu}}(\mathbf{NC}))\right)}{\left(k_{\text{rev}}^{-\text{Tu}}(\mathbf{C}) + k_{\text{CCA}}\right) \left(k_{\text{EA}}^{+\text{Tu}}(k_{\text{CCA}} + k_{-\text{Tu}}) + k_{-\text{Tu}}(k_{\text{CCA}} + k_{-\text{Tu}} + k_{\text{rev}}^{+\text{Tu}}(\mathbf{C}))\right)} \quad (14)$$

$$P_{\text{f}}^{\text{EA}} = \left(\frac{1 + \frac{k_{-\text{Tu}}}{k_{\text{rev}}^{-\text{Tu}}(\mathbf{C}) + k_{\text{CCA}}}}{1 + \frac{k_{-\text{Tu}}}{k_{\text{rev}}^{-\text{Tu}}(\mathbf{NC}) + k_{\text{CCA}}}} \right) \left(\frac{1 + \frac{k_{-\text{Tu}} k_{\text{rev}}^{+\text{Tu}}(\mathbf{NC})}{(k_{\text{EA}}^{+\text{Tu}} + k_{-\text{Tu}})(k_{\text{CCA}} + k_{-\text{Tu}})}}{1 + \frac{k_{-\text{Tu}} k_{\text{rev}}^{+\text{Tu}}(\mathbf{C})}{(k_{\text{EA}}^{+\text{Tu}} + k_{-\text{Tu}})(k_{\text{CCA}} + k_{-\text{Tu}})}} \right) \quad (15)$$

The proofreading factor factors into two terms, consisting of contributions containing $k_{\text{rev}}^{+\text{Tu}}$ and $k_{\text{rev}}^{-\text{Tu}}$. Note that, as $k_{-\text{Tu}} \to 0$, $P_{\text{f}}^{\text{EA}} \to 1$. So if EF-Tu never dissociates, there can be no

proofreading because rejection would not be allowed. In the limit of $k_{\mathrm{EA}}^{+\mathrm{Tu}} \to \infty$:

$$P_{\mathrm{f}}^{\mathrm{EA}} = \frac{1 + \frac{k_{-\mathrm{Tu}}}{k_{\mathrm{rev}}^{-\mathrm{Tu}}(\mathrm{C}) + k_{\mathrm{CCA}}}}{1 + \frac{k_{-\mathrm{Tu}}}{k_{\mathrm{rev}}^{-\mathrm{Tu}}(\mathrm{NC}) + k_{\mathrm{CCA}}}} \tag{16}$$

Since the factor depending on $k_{\mathrm{rev}}^{+\mathrm{Tu}}$ in Eq. 15 does not vary significantly (1 to 2) for reasonable values of the parameters, the more influential factor is the first term. In the additional limit of $k_{\mathrm{CCA}} \to \infty$:

$$P_{\mathrm{f}}^{\mathrm{EA}} = \frac{1 + \frac{k_{-\mathrm{Tu}}}{k_{\mathrm{rev}}^{-\mathrm{Tu}}(\mathrm{C}) + \infty}}{1 + \frac{k_{-\mathrm{Tu}}}{k_{\mathrm{rev}}^{-\mathrm{Tu}}(\mathrm{NC}) + \infty}} \tag{17}$$

$$= 1 \tag{18}$$

The lack of proofreading in this limit demonstrates why accommodation must have a slow step. Taking the simultaneous limits $k_{-\mathrm{Tu}} \to \infty$ and $k_{\mathrm{CCA}} \to 0$ gives $P_{\mathrm{f}}^{\mathrm{EA}} = k_{\mathrm{rev}}^{-\mathrm{Tu}}(\mathrm{NC})/k_{\mathrm{rev}}^{-\mathrm{Tu}}(\mathrm{C}) \equiv P_{\mathrm{f,max}}$. $P_{\mathrm{f,max}}$ is the theoretical maximum proofreading given the differential reverse rate. As described below, under the same limit that gives $P_{\mathrm{f,max}}$, the efficiency goes to zero.

The efficiency E of proofreading can be quantified by the number of cognate tRNA that are accepted relative to the total tRNA that make it past initial selection. We again apply the condition that $k_{\mathrm{reject}} \gg k_{\mathrm{EA}}^{-\mathrm{Tu}}$.

$$E = \frac{d[\mathrm{A}^{\mathrm{C}}]/dt}{d[\mathrm{A}^{\mathrm{C}}]/dt + d[\mathrm{A/T}^{-\mathrm{Tu}\ \mathrm{C}}]/dt} \tag{19}$$

$$= \frac{k_{\mathrm{EA}}^{+\mathrm{Tu}} k_{\mathrm{CCA}} (k_{\mathrm{CCA}} + k_{-\mathrm{Tu}} + k_{\mathrm{rev}}^{-\mathrm{Tu}}(\mathbf{C}))}{(k_{\mathrm{rev}}^{-\mathrm{Tu}}(\mathbf{C}) + k_{\mathrm{CCA}}) \left(k_{\mathrm{EA}}^{+\mathrm{Tu}}(k_{\mathrm{CCA}} + k_{-\mathrm{Tu}}) + k_{-\mathrm{Tu}}(k_{\mathrm{CCA}} + k_{-\mathrm{Tu}} + k_{\mathrm{rev}}^{+\mathrm{Tu}}(\mathbf{C}))\right)} \tag{20}$$

Supplementary References

[1] Nguyen, K. & Whitford, P. C. Steric interactions lead to collective tilting motion in the ribosome during mRNA-tRNA translocation. *Nat Comm* **7**, 10586–10586 (2016).

[2] Noel, J. K., Chahine, J., Leite, V. B. P. & Whitford, P. C. Capturing transition paths and transition states for conformational rearrangements in the ribosome. *Biophys. J.* **107**, 2881–2890 (2014).

[3] Jenner, L. B., Demeshkina, N., Yusupova, G. & Yusupov, M. Structural aspects of messenger RNA reading frame maintenance by the ribosome. *Nat. Struct. Mol. Biol.* **17**, 555–560 (2010).

[4] Schmeing, T. M., Voorhees, R. M., Kelley, A. C. & Ramakrishnan, V. How mutations in tRNA distant from the anticodon affect the fidelity of decoding. *Nat. Struct. Mol. Biol.* **18**, 432–436 (2011).

[5] Polekhina, G. *et al.* Helix unwinding in the effector region of elongation factor EF-Tu-GDP. *Structure* **4**, 1141–1151 (1996).

[6] Hess, B., Kutzner, C., van der Spoel, D. & Lindahl, E. GROMACS 4: Algorithms for highly efficient, load-balanced, and scalable molecular simulation. *J. Chem. Theory Comput.* **4**, 435–447 (2008).

[7] Noel, J. K. *et al.* SMOG 2: A versatile software package for generating structure-based models. *PLoS Comp Biol* **12**, e1004794 (2016).

[8] Whitford, P. C. *et al.* Accommodation of aminoacyl-tRNA into the ribosome involves reversible excursions along multiple pathways. *RNA* **16**, 1196–1204 (2010).

[9] Garcia, A., Krumhansl, J. & Frauenfelder, H. Variations on a theme by Debye and Waller: From simple crystals to proteins. *Proteins: Struct., Funct., Bioinf.* **29**, 153–160 (1997).

[10] Hayes, R. L. *et al.* Reduced model captures Mg(2+)-RNA interaction free energy of riboswitches. *Biophys. J.* **106**, 1508–1519 (2014).

[11] Ferrenberg, A. & Swendsen, R. Optimized Monte Carlo data analysis. *Phys. Rev. Lett.* **63**, 1195–1198 (1989).

[12] Bryngelson, J. D. & Wolynes, P. G. Intermediates and barrier crossing in a random energy model (with applications to protein folding). *J. Phys. Chem.* **93**, 6902–6915 (1989).

[13] Whitford, P. C., Onuchic, J. N. & Sanbonmatsu, K. Y. Connecting energy landscapes with experimental rates for aminoacyl-tRNA accommodation in the ribosome. *J. Am. Chem. Soc.* **132**, 13170–13171 (2010).
